# Molecular identification of spiders preying on *Empoasca vitis* in a tea plantation

**DOI:** 10.1038/s41598-017-07668-w

**Published:** 2017-08-10

**Authors:** Ting-bang Yang, Jie Liu, Long-yu Yuan, Yang Zhang, Dai-qin Li, Ingi Agnarsson, Jian Chen

**Affiliations:** 1 Centre for Behavioural Ecology and Evolution (CBEE), and Hubei Collaborative Innovation Center for Green Transformation of Bio-Resources, College of Life Sciences, Hubei University, Wuhan, 430062 China; 2 Department of Biology, University of Vermont, Burlington, VT USA; 3 Department of Biological Sciences, National University of, Singapore, Singapore

## Abstract

Biological control using predators of key pest species is an attractive option in integrated pest management (IPM). Molecular gut analysis can provide an estimation of predator efficiency on a given prey. Here we use a combination of various experimental approaches, both in field and lab, to identify a potential biological control species of the common pest of commercially grown tea, *Empoasca vitis* (Göthe) (Hemiptera), in a Chinese plantation. We collected 2655 spiders from plantations and established relative abundances of spider species and their temporal overlap with the pest species in tea canopy. We analyzed DNA from 1363 individuals of the most common spider species using targeted RQ-PCR to quantify the potential efficiency of spiders as a predator on *E*. *vitis*. The results showed that, in the field, the jumping spider *Evarcha albaria* was the most abundant, had the closest temporal overlap with the pest, and frequently fed on it. Therefore, this spider may play a key role in pest suppression. The present study demonstrates the potential of our experimental approach to study predator-prey relationships in taxa that do not lend themselves to morphological identification of gut contents, such as spiders.

## Introduction

Tea, as one of the world’s most popular beverages and is an important cash crop that is cultivated worldwide^1–4^, with China, as the largest current producer^4^. Its popularity, in part, stems from its richness in polyphenols that may have various health benefits such as reduce risk of cancer^2, 5^. The yield and quality of tea from plantations is negatively impacted by several tea pest species, putting a premium on effective means of control^6^. Direct applications of pesticides applied to tea is the most widely used and effective pest control^6^, however, this method has a series of negative side effects, including pesticide residues, pesticide resistance in important pest species, damage to natural pest enemies, and others^7–9^. Hence, emphasis on biological control is increasing, focusing on the preservation and introduction of natural enemies like predators, parasitoids and pathogens for suppression of pests to commercially tolerable levels^6, 10^.

Spiders are among the most abundant predators of insects in terrestrial ecosystems^11^. Nyffeler & Birkhofer^12^ have estimated that the annual prey kill of the global spider community is in the range of 400–800 million metric tons. In tea plantations, spiders, ladybird beetles, assassin bugs, lacewings, and praying mantises, are the predominant predators on pest species^13^. Spiders, in particular, play key functional role. They are the most abundant predators with dominance ranging from 65.0% to 97.8% in Chinese tea plantations^14^. Spiders have been shown to play an important role in biological control through analyses of population dynamics between the spiders and pests in the field^15–20^. However, field evidence is often lacking for if, and how effectively, spiders prey on the pest of concern. Detailed studies of the actual prey-predator interactions between the spiders and the pests and efforts to quantify predator efficiency are thus important to better understand the roles spiders might play in biological pests control^21, 22^. The dominance of predators, temporal and spatial overlap between predators and pests, predation rates, and prey numbers consumed per predatory individual, are often used as indicators of which predators control pests^23–28^. One of the challenges here is to identify prey post-predation. Spiders liquefy their food externally before ingestion, ruling out morphological identification of gut contents. PCR methodology has been shown to be useful in detecting the presence prey species in spider guts^29^, however, this does not allow good estimation of predator efficiency on a given prey. Real-time quantitative PCR (RQ-PCR) offers additional information on the abundance of target DNA of prey tissue digested in predators’ gut and thus can yield an estimate of relative importance of predators^29^.

As discussed in King *et al*.^29^, RQ-PCR can quantify single target prey species DNA copies in predator guts, using a single primer. The predation rates and prey numbers consumed per predatory individual are then estimated by the positive rates and copy numbers of target fragment of prey remains in the gut of predators, respectively, offers a qualitative assessment of predation in the field. To date, TaqMan RQ-PCR has already been used to amplify and quantify target DNA of the prey remains in predators’ gut^25, 28, 30, 31^. Using a pair of primers and a fluorogenic oligodeoxy-nucleotide probe, which is specific for the target prey, the target DNA of the prey remains in predators’ gut can be quantified. Short prey amplicons (usually <300 bp) are optimal to target the already fragmented DNA ‘post digestion’ in the predator^32, 33^. The mitochondrial cytochrome oxidase subunit I (mtCOI) has proven useful as a marker due to the abundance of copies of this gene, species specificity, and richness of data available in public databases^29^.

*Empoasca vitis* (Göthe) (Hemiptera) is one of the most damaging tea pests in China that can seriously decrease tea yield by sucking juices out of the tender tea-leaf directly^34–36^. Gao *et al*.^37^ studied the predation of *Evarcha albaria* (L. Koch) (Araneae) on *E*. *vitis* under laboratory conditions. The results showed that both male and female spiders preyed on the nymph and adult of *E*. *vitis*. However, the role of spiders as predator on *E*. *vitis* in the field is unknown. Spiders are highly variable in hunting strategies, habitat preferences, and activity periods, which combine to make the group potentially highly efficient in controlling certain groups of pests^16^. In tea plantations, many spider species hunt prey in the canopies of tea^38^, indicating relatively high spatial-sympatry with the tea pests. Here we aim to screen the main predators of *E*. *vitis* in the canopies of tea, testing the spider species as potential biological control agents of *E*. *vitis*. Therefore, the present study focus mainly on three aspects: (1) establishing the relative abundances of spider species; (2) determining the temporal overlap between dominant spider species and the pest species in tea canopy; (3) providing an approximately quantitative assessment of relative efficiency of spiders as a predator on *E*. *vitis* using RQ-PCR.

## Results

### The abundances, population fluctuations, and temporal overlap of dominant spider species and *E*. *vitis* in the field

**Figure 1:**
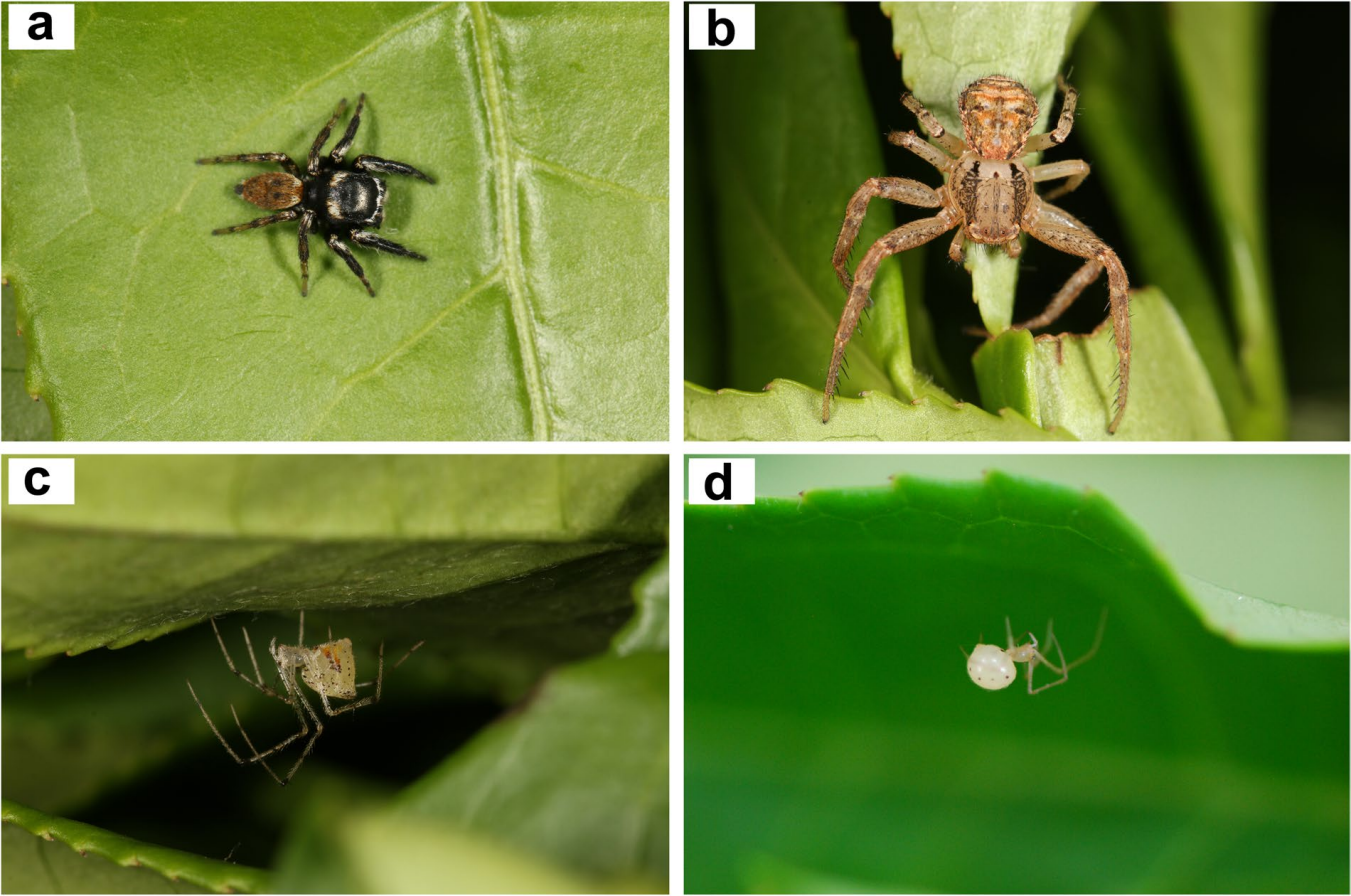
Four dominant spider species in the canopies of tea in the study area. (**a**) *E*. *albaria* (male); (**b**) *X*. *ephippiatus* (female); (**c**) *M*. *pulcherrima* (female); (**d**) *C*. *octomaculatum* (female). Photos taken by first author.

**Figure 2:**
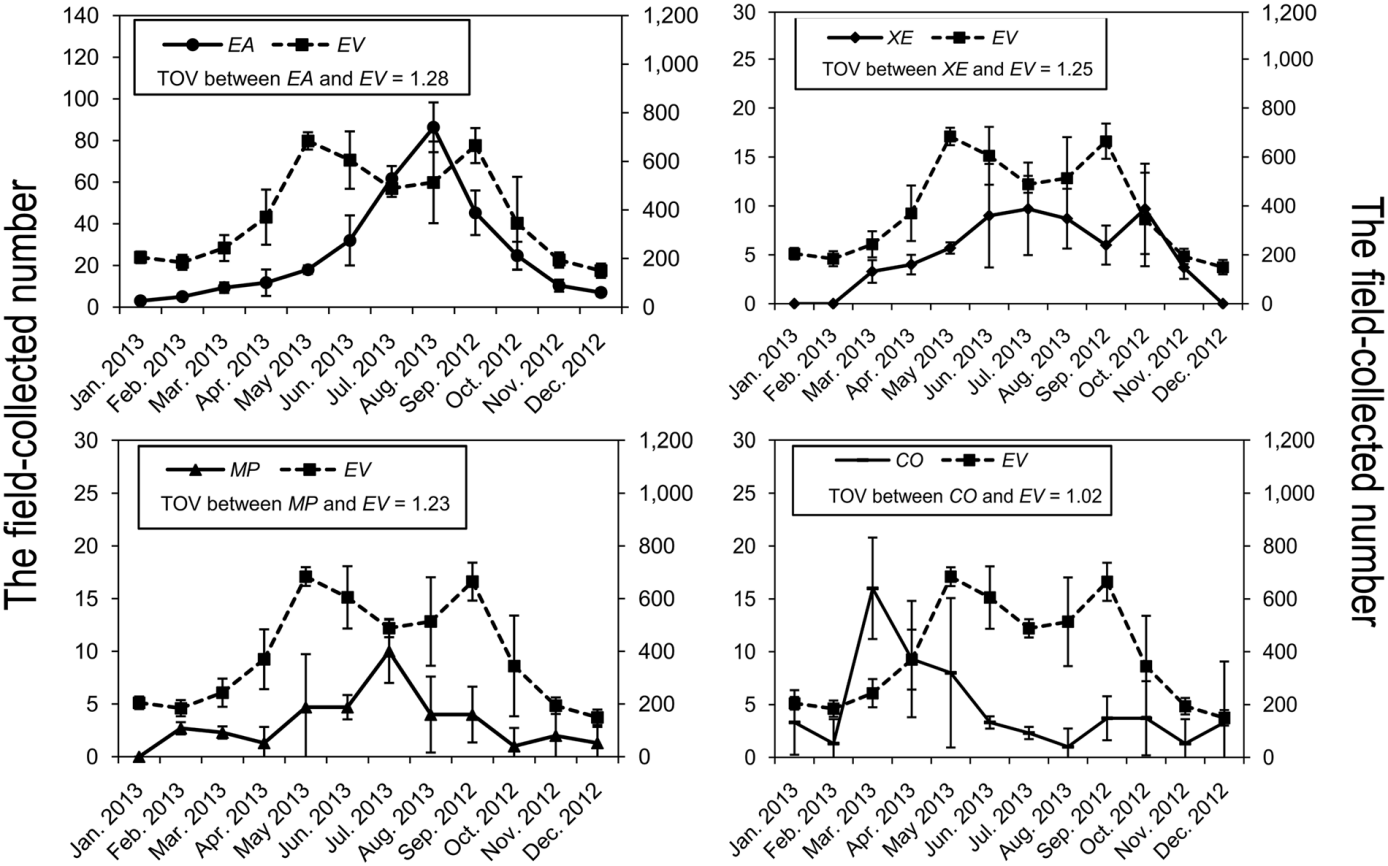
Population dynamics of *E*. *vitis* and four dominant spider species in the canopies of tea. Values presented as mean ± SD (N = 3). Temporal overlap values (TOV) between spider species and *E*. *vitis* was estimated by following measure, developed by Hurlbert^61^: ${Lij}={S}{\sum }_{h=1}^{S}{P}_{ih}{P}_{jh}$; where, *L*_*ij*_ is the temporal and spatial overlap measure of species *i* on species *j*, *S* is the unit number of resource sequence, *P*_*ih*_ is the proportion that resource *h* is of the total resource that species *i* utilizes and *P*_*jh*_ is the proportion that resource *h* is of the total resource that species *j* utilizes. *E*. *vitis*: *EV*; *E*. *albaria*: *EA*; *X*. *ephippiatus*: *XE*; *M*. *pulcherrima*: *MP*; *C*. *octomaculatum*: *CO*.

In total 2655 (including 885 adults and 1770 juveniles) individual spiders were collected belonging to 50 species. Among them, *Evarcha albaria* (L. Koch) (Salticidae), *Xysticus ephippiatus* Simon (Thomisidae), *Meotipa pulcherrima* (Mello-Leitão) (Theridiidae) and *Coleosoma octomaculatum* (Bösenberg & Strand) (Theridiidae) (Fig. 1) were the most dominant in the canopies of tea with relative adult abundances of 43.2%, 10.7%, 8.5% and 8.4%, respectively (Table S1). We focus on these four spider species in further analyses. Abundances of dominant spider species (*E*. *albaria*, *X*. *ephippiatus*, *M*. *pulcherrima* and *C*. *octomaculatum*) and the pest *E*. *vitis* are shown in Fig. 2 and Table S2. Abundances show seasonal fluctuation with *E*. *vitis* being most abundant from May to September, *E*. *albaria* from May to October, *X*. *ephippiatus* from June to October, *M*. *pulcherrima* from May to September and *C*. *octomaculatum* from March to May. The temporal overlap values between spider species and *E*. *vitis* was 1.28, 1.25, 1.23 and 1.02, respectively. The largest overlap values are between *E*. *albaria* and *E*. *vitis*.

### Specificity of primers

The primers (ZJ-F and ZJ-R), using TaqMan RQ-PCR, successfully amplified *E*. *vitis* DNA (146 bp), but did not amplify predators, 16 other potential prey species, or tea-leaf (Table 1).

**Table 1:** The species which was chosen for cross reactivity to the assay.

Order	Family	Species
Hemiptera	Aphididae	*Toxoptera aurantii*
	Coccidae	*Ceroplastes* sp.
	Aleyrodidae	*Aleurocanthus* sp.
	Miridae	*Lygocoris sp*.
		*Cyrtorrhinus* sp.
Lepidoptera	Geometridae	*Ectropis obliqua*
		*Scopula* sp.
		*Buzura* sp.
	Lymantriidae	*Euproctis pseudoconspersa*
	Gracillariidae	*Caloptilia* sp.
	Pieridae	*Pieris rapae*
Coleoptera	Curculionidae	*Myllocerinus aurolineatus*
	Elateridae	*Agriotes* sp.
Diptera	Syrphidae	*Syrphus* sp.
Orthoptera	Tettigoniidae	*Holochlora nawae*
Thysanoptera	Thripidae	*Scirtothirips* sp.

### Standard curve, sensitivity and reproducibility of TaqMan RQ-PCR

The sensitivity of the assay was evaluated using 10-fold serial dilution of all stock plasmids ranging from 1.92 × 10^9^ to 1.92 × 10° copies/µL. The result showed that the test could detect as few as 192 copies of the target-fragment (Table 2) and there was a strong linear relationship between the *Ct* values and the log_10_ of the input number of copies (*R* ^2^ = 0.9992). The slope and intercept of standard curve was −3.34 and 45.37, respectively. Amplification efficiency was 97.6% (Fig. S1). Evaluating reproducibility of the assay between three sub-samples, the coefficients of variation were <5% (0.20–1.39%), which indicated that the assay was highly reliable and reproducible (Table 2).

**Table 2:** The sensitivity and reproducibility of TaqMan RQ-PCR.

CNTF	*Ct*	CV (%)
1.92 × 10^9^	14.20 ± 0.10	0.70
1.92 × 10^8^	17.25 ± 0.24	1.39
1.92 × 10^7^	20.51 ± 0.14	0.68
1.92 × 10^6^	23.80 ± 0.12	0.50
1.92 × 10^5^	27.40 ± 0.20	0.73
1.92 × 10^4^	30.33 ± 0.06	0.20
1.92 × 10^3^	33.39 ± 0.15	0.45
1.92 × 10^2^	37.29 ± 0.09	0.24
1.92 × 10^1^	—	—
1.92 × 10^0^	—	

Values presented as mean ± SD (N = 3). The assay was evaluated using 10-fold serial dilution of all stock plasmids ranging from 1.92 × 10^9^ to 1.92 × 10^0^ copies/µL. *Ct* stands for RQ-PCR cycle number where fluorescence curve crosses threshold line. CV stands for coefficients of variation.

### Efficiency of molecular marker in detecting *E*. *vitis* remains in spider guts

TaqMan RQ-PCR was employed to qualitatively and quantitatively detect the genomic DNA of four adult spider species at various time periods after consuming one adult *E*. *vitis*. The results show that all spiders that consumed prey were positive for *E*. *vitis* DNA at *t* = 0 h, decreasing to 60% (*E*. *albaria*), 0 (*X*. *ephippiatus*), 60% (*M*. *pulcherrima*) and 40% (*C*. *octomaculatum*) after 60 h of digestion. After 72 h of digestion, the positive rate decreased to 0 (Table 3). The CNTF (copy numbers of target fragment) of 0 hour digestion was 1.23 × 10^7^ ± 9.88 × 10^5^ (*E*. *albaria*), 9.77 × 10^6^ ± 2.55 × 10^6^ (*X*. *ephippiatus*), 1.49 × 10^7^ ± 7.01 × 10^6^ (*M*. *pulcherrima*) and 4.50 × 10^7^ ± 3.07 × 10^6^ (*C*. *octomaculatum*).

**Table 3:** Detection efficiency in adult spider individuals at various time after feeding on one adult *E*. *vitis* using TaqMan RQ-PCR.

Hours post-feeding (h)	Number of samples	The PRTF (%)			
		*E*. *albaria*	*X*. *ephippiatus*	*M*. *pulcherrima*	*C*. *octomaculatum*
0	5	100	100	100	100
12	5	100	100	100	100
24	5	100	100	100	100
36	5	100	100	100	100
48	5	100	60	100	100
60	5	60	0	60	40
72	5	0	0	0	0

### Qualitative and quantitative evaluation of predation in the field

Genomic DNA of all spiders (including juvenile and adult) were detected by TaqMan RQ-PCR. The results show that the PRTF (positive rates of target fragment) in spiders gut was 54.2% (*E*. *albaria*, N = 943, male: 160, female: 222, juvenile: 561), 30.1% (*X*. *ephippiatus*, N = 176, male: 25, female: 70, juvenile: 81), 17.5% (*M*. *pulcherrima*, N = 114, male: 24, female: 50, juvenile: 40) and 4.6% (*C*. *octomaculatum*, N = 130, male: 28, female: 47, juvenile: 55), and it showed significant differences between those of *E*. *albaria* and any other species (Table 4). Correspondingly, the average of residual minimum number of *E*. *vitis* in individual spider gut was 0.63 ± 0.05, 0.05 ± 0.02, 0.04 ± 0.02 and 0.02 ± 0.01, respectively, and it also showed significant differences between those of *E*. *albaria* and any other species (Table 4).

**Table 4:** The PRTF (positive rates of target fragment) and residual minimum number of *E*. *vitis* in per dominant spider species.

Species	Field-collected number (juvenile and adult)	The PRTF (%)	The average of residual minimum number of *E*. *vitis* in individual spider guts
*E*. *albaria*	943	54.2a	0.63 ± 0.05a
*X*. *ephippiatus*	176	30.1b	0.05 ± 0.02b
*M*. *pulcherrima*	114	17.5c	0.04 ± 0.02b
*C*. *octomaculatum*	130	4.6 d	0.02 ± 0.01b

Values presented as mean ± SE. Residual minimum number of *E*. *vitis* in individual spider guts = *N*/*M* (*N* is the CNTF (copy numbers of target fragment) of field-collected individual spiders; *M* is the CNTF of individual spiders which feeding one adult *E*. *vitis* after 0 hour digestion). The different lowercase in same column indicate significant difference in *P* < 0.05 level.

**Figure 3:**
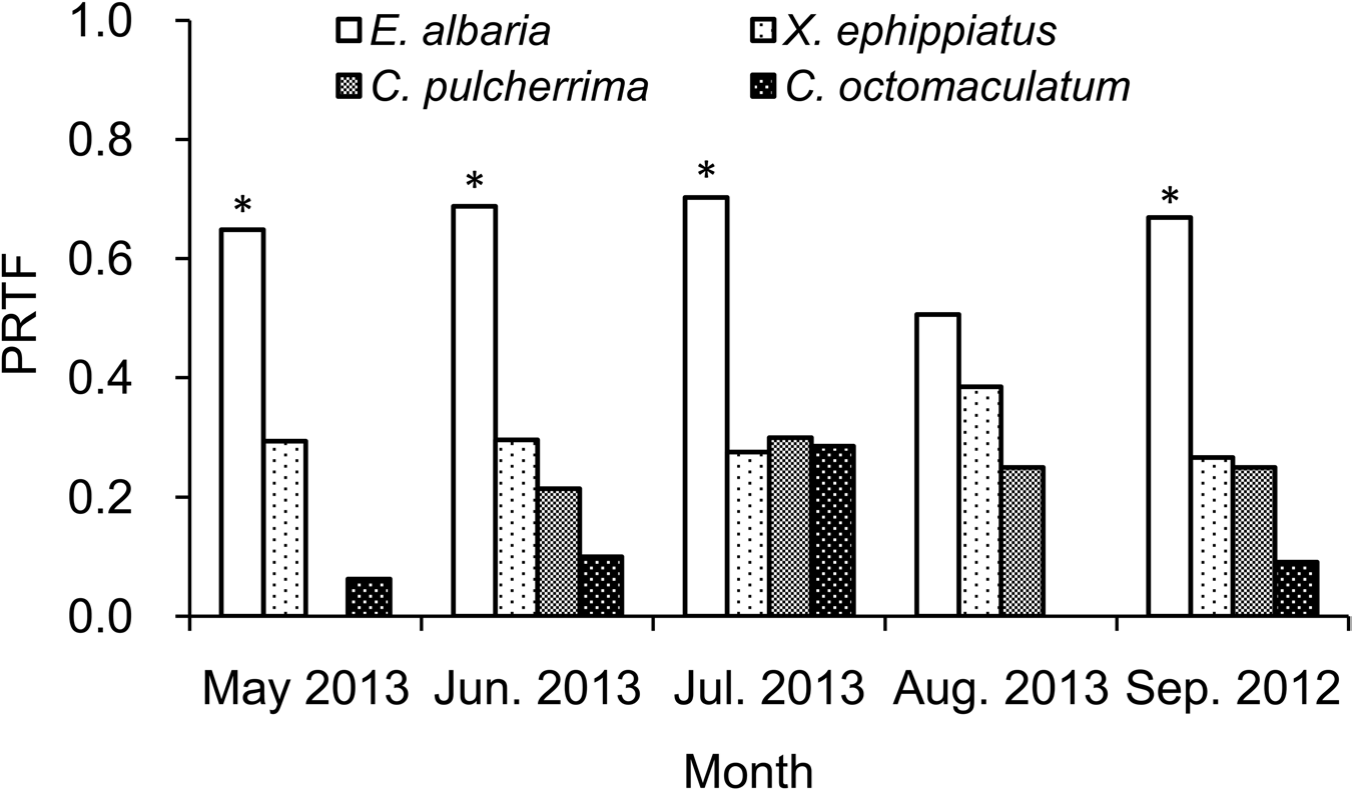
The PRTF (positive rates of target fragment) in spiders gut during the months when *E*. *vitis* was abundant. *In figure indicate significant difference in *P* < 0.05 level between *E*. *albaria* and any other species. The same below.

**Figure 4:**
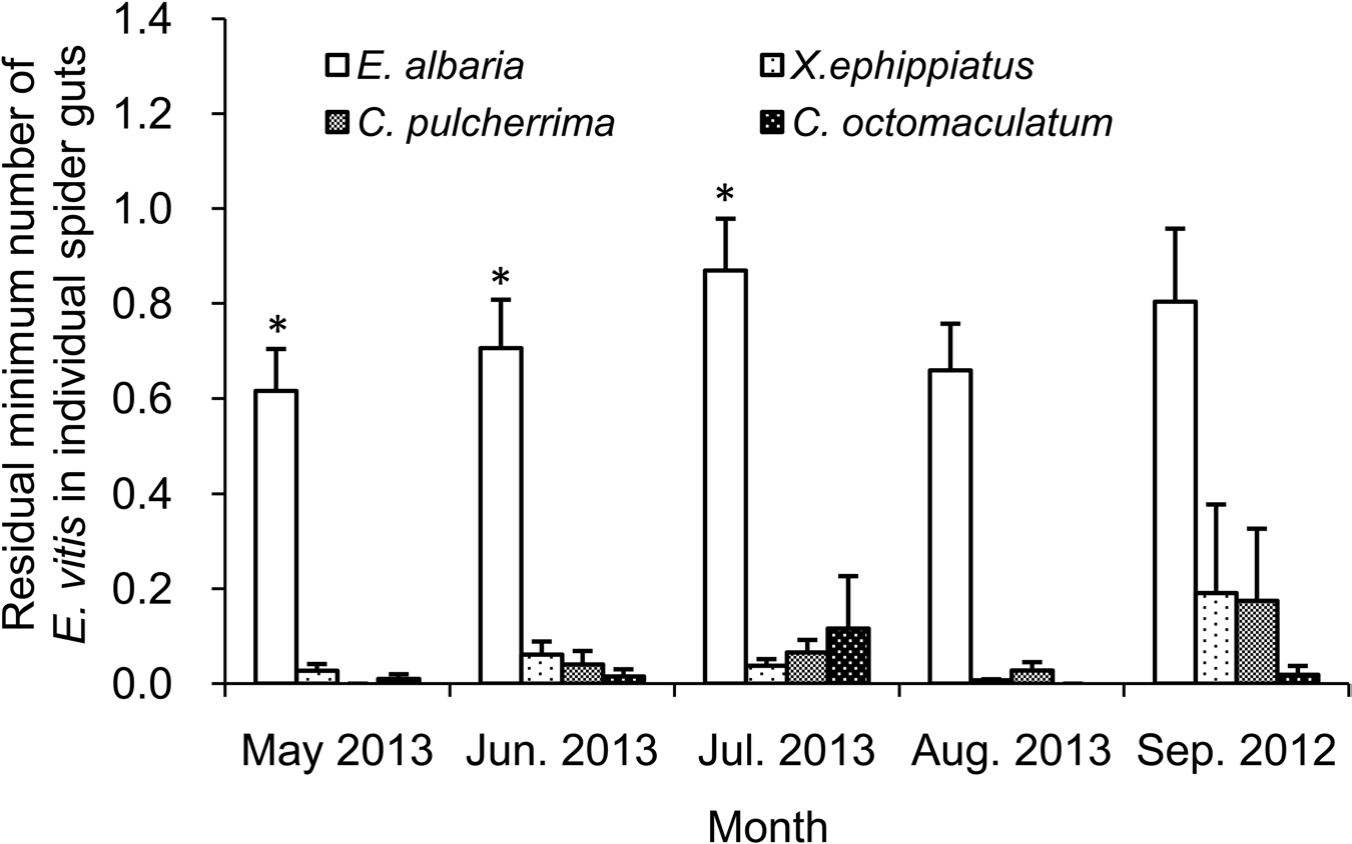
The residual minimum number of *E*. *vitis* in individual spider gut during the months when *E*. *vitis* was abundant.

As shown in Figs 3 and 4, the PRTF in *E*. *albaria* guts was significantly greater than those of any other predators, during the months when *E*. *vitis* was most abundant in, May, June, July and September. The residual minimum number of *E*. *vitis* in *E*. *albaria* guts was also greater than those of any other predator, significantly so in May, June, and July.

## Discussion

Tea is an important commercial crop in China impacted by a variety of pest species, with one of the most common being the true bug *E*. *vitis* (Hemiptera). Effective control of this pest has long been achieved via chemical pest control^6, 9^. However, the negative side effects of chemical use put a premium on establishing viable alternative pest control approaches. Biological control using natural enemies of pest species is an attractive option but relies on identifying the interactions between the pest and the potential control agent. Previous studies have indicated a jumping spider (*E*. *albaria*, Salticidae) as a dominant spider in tea plantations of China^39–42^, and laboratory experiments have demonstrated that it feeds on *E*. *vitis* ^37^. However, data on field predation efficiency are more challenging to obtain, not the least in predator species that predigest prey outside the body, such as spiders. Thus, we lack an understanding of these species interactions in the more complex food web in the field, and therefore the fundamental basis for establishing the potential of *E*. *albaria* as a biological control agent.

We performed analyses of gut contents from field-collected spiders using a combination of various experimental approaches both in field and lab based on 2655 spiders from plantations, and established relative abundances of spider species and their temporal overlap with the pest species. DNA analyzed from 1363 individuals of the most common spider species using targeted RQ-PCR clearly show that the jumping spider *E*. *albaria*—found to be the most abundant spider in the field and the one with the closest temporal overlap with the pest in tea canopy—was the most effective predator on *E*. *vitis*. Pest DNA was amplified from 54.2% of these spiders with a minimum of 0.63 ± 0.05 *E*. *vitis* individuals per *E*. *albaria* gut from the field, despite limits of detection success beyond 72 hrs after feeding. Relatively high feeding rates combined with high spider abundance, and temporal overlap and spatial-sympatry between the predatory natural enemies and the prey all are indicators of a potentially effective biological control species^27, 43, 44^. Therefore, we conclude that *E*. *albaria* is a key predator of *E*. *vitis* and that it has potential as a biological control agent of *E*. *vitis*. To evaluate the potential effectiveness of *E*. *albaria* as a component in an IPM strategy, however, further experimental research is necessary. Most urgently needed are experiments with selective exclusion—or population enhancements—of the predator species.

To date, there are many methods (including direct observation^45^, gut dissection^46^, radioisotope labeling^47^, monoclonal antibodies^48, 49^ and molecular gut analysis^29^) that can be employed to identify the interactions between predators and prey in the field. Among them, molecular gut analysis and monoclonal antibodies are especially practical for studying predation of some relatively small arthropod predators in the field. Our approach can qualitatively assess predation in the field, however, like monoclonal antibodies, our method cannot accurately estimate the number of the prey consumed by the predator per time unit^29, 50^, and is therefore not directly quantitative.

Our estimate of predator efficiency is limited by this difficulty of quantifying the number of prey consumed per predator, as well as by the decay of prey DNA in predator gut (0 detection rate after 72 hours). However, our approach allows at least a relative estimate of prey numbers per spider species. The more prey consumed, the more CNTF remains in the gut of the spiders^25^. We quantitatively detected the target fragment of *E*. *vitis* remains in the gut of four dominant spider species in tea plantation, and compared the residual minimum number of *E*. *vitis* in individual spider guts. We found that the average of residual minimum number of *E*. *vitis* in *E*. *albaria* gut (0.63 ± 0.05) was significantly greater than those of any other common spider species. The number of prey DNA copies in spider guts result from an interplay of ambient temperature, prey number, prey size, and time since ingestion^51^ and thus these estimates are approximate. However, while our methods do not establish the number of consumed prey, they do allow a meaningful comparison of the relative importance of various predators by comparing the residual minimum number of *E*. *vitis* in the guts of individual predators. We find clear statistical differences in the average of residual minimum number of *E*. *vitis* in individual spider guts, indicating differences in number of prey, and these numbers are highest for our targeted biological control species. Clearly then, the consumption of this prey is a frequent and likely important part of the *E*. *albaria* daily diet. In turn, frequent predation on the pest by majority of prey individuals suggests that the spider is a significant predator of *E*. *vitis* meriting further research of its potential as a part of an IPM^52^.

PCR is a sensitive method for gene amplification, even capable of amplification from a single target molecule^53^. It is, however, susceptible to many interference factors, such as PCR inhibitors, that can lead to amplification-failure^29^. Design of modern DNA extraction and purification kits have greatly reduced this problem and furthermore, simply diluting the concentration of the DNA template has proven to be an easy and effective approach to overcoming PCR inhibition^29^. Our experiments have also demonstrated that diluting the concentration of the DNA template could effectively overcome the occurrence of the false-negatives (Fig. S2). Wang *et al*.^28^ had detected the effect of different background DNA of predators by using target prey DNA diluted in water and in the genomic DNA mixture of starved predator species. The result indicated that background DNA does not affect amplification. Our experiments have also demonstrated that different background DNA of four dominant spider species does not affect amplification of target fragment of *E*. *vitis* (Table S3). This suggests the current RQ-PCR protocol may be very effective in target identification of gut contents. Additionally, in order to ensure the fidelity and reliability of the assay, each DNA sample should assay in triplicate^54^, and a non-template control (without any nucleic acid), a negative control (without target DNA), and a positive control (including target DNA), should be contained in each PCR run. In this study, three sub-samples yielded the consistent results, indicating that the assay is highly reliable and reproducible.

Determining the relationship between predator and prey and the potential role a predator may play in pests control in the field is important^55, 56^. It is also significant studying the trophic niche of predators as they can be feeding on multiple types of prey. Molecular approaches provide useful tools for determining the dietary breadth of such predators. Petráková *et al*.^57^ has studied on the trophic niche of *Ammoxenus amphalodes* (Araneae, Ammoxenidae) using Next Generation Sequencing (NGS), and revealing that *A*. *amphalodes* is a specialist termite-eating spider, only capturing *Hodotermes mossambicus* (Isoptera, Hodotermitidae) as its food. Such approaches using NGS would be the natural next step in determining the dietary breadth of the main predators identified in Chinese crop fields.

## Methods

### Collection of samples

The study was carried out in Wang Dazhen tea plantation which is located near Xianning city, Hubei province, China (114.352°E, 29.953°N). *E*. *vitis* is a dominant species of tea pests, in this area. The total area of the studied tea plantation is about 6.5 ha. with parallel rows of tea plants about 100 m long and 1 m apart. During the sampling period, no insecticide was applied in the tea plantation.

The spiders and pest were collected on dry days, and each sampling period lasted at least seven days (three times a month), with the same person in tea plantation from September 2012 to August 2013. A total of 30 transects, separated by at least 10 m were sampled every time, with three sampling sites randomly chosen at each transect separated by at least 15 m. A total of 90 sites were randomly collected each sampling period using an insect net (diameter: 40 cm) under tea canopies, and beating the canopies ten times with a stick. The number of *E*. *vitis* was counted, and all spiders were individually put in 1.5 mL microcentrifuge tubes with 100% ethanol, kept on ice until returning to the laboratory and later stored at −80 °C.

Spiders were identified from the reference keys and catalogues provided by Yin *et al*.^58^ and World Spider Catalog^59^.

### DNA extraction

The genomic DNA of spiders was subsequently extracted and purified individually using an animal genomic DNA extraction kit (Beijing Dingguo Changsheng Biotechnology Co., Ltd., Beijing, China) according to the manufacturer’s instructions. Finally, The DNA was resuspended in 100 µL of manufacturer’s elution buffer and stored at −80 °C.

### Design of primers and TaqMan minor groove binder (MGB) probe

We followed the rules on species-specific primer design outlined by King *et al*.^34^. Ideal primers should be efficient at high annealing temperatures (if possible, *T*a > 55 °C), thus reducing the risk of nonspecific amplification. In addition, shorter fragments <300 bp should be targeted wherever possible as the DNA molecules are broken into smaller fragments during digestion in the predator guts. We used a fragment of the COI gene of *E*. *vitis* from GeneBank (named ZJ5 isolate, 773 bp, GeneBank Accession no. KC172507.1) to design the primer pair ZJ-F: 5′-AGGTGCTGTATTTGCTAT-3′ and ZJ-R: 5′-CTAAGAAATGTTGAGGGA-3′, which amplify a 146 bp fragments, using Gene tool software.

The TaqMan MGB probe for the *E*. *vitis* target DNA quantification was designed by using the Primer Express v3.0 software. The probe (5′-TTACCCCAAAGAATATCAC-3′), hybridized within the region amplified by the PCR primers from base 507 to base 525 of ZJ5 isolate, and synthesized by Shanghai Bioligo Biotechnology Ltd. (Shanghai, China), at the 5′-end there is a reporter [FAM (6-carboxy-fluorescein)] and at the 3′-end a minor groove binding (dehydrocyclopyrroindole tripetide, DPI3).

### The specificity of primers

Our goal was to detect the specific DNA fragments of *E*. *vitis* remains in the gut of generalist-predators spiders. Therefore, to confirm that the developed primers (ZJ-F and ZJ-R) can specifically amplify DNA fragments of *E*. *vitis* remains in the gut of spiders, we performed cross-reactivity assays with the primers (ZJ-F and ZJ-R) using the various prey species. Species specificity of the primers was tested using genomic DNA from target prey, predator and potential prey species and tea leaf. The potential prey species of spiders and tea leaf were collected in tea plantation. The genomic DNA of individual target prey, predator and potential prey species and tea leaf (50 mg) were amplified simultaneously by the primers (ZJ-F and ZJ-R).

### The standard curve, sensitivity and reproducibility of TaqMan RQ-PCR

The purified target-fragment of *E*. *vitis* were ligated and cloned into PUC57 vector (Shanghai Bioligo Biotechnology Co., Ltd., Shanghai, China), propagated in DH5a competent cells (Beijing TransGen Biotechnology Co., Ltd., Beijing, China). The recombinant plasmids DNA were isolated from the obtained white colonies and inoculated in liquid medium (LB/Amp). The recombinant plasmids DNA were purified by using an AxyPrep Plasmid Miniprep Kit (Axygen Biosciences, USA) according to the manufacturer’s instructions. The DNA was eluted in 50 μL eluent and stored at −80 °C. To confirm whether the inserted DNA is consistent with the target DNA, the recombinant plasmids DNA was sequenced by Wuhan TsingKe Biological Technology Co., Ltd. (Wuhan, China). Its concentration (ng/µL) was determined by spectrophotometric measurement (NanoDrop 2000c, Thermo Fisher Scientific Inc, USA) and the copy numbers of target fragment (CNTF) were calculated using the expression: CNTF = [DNA mass (g)/recombinant plasmid molarmass] × 6.02 × 10^23^.

The recombinant plasmid was used as standards for calibration of copy number. The number of transcripts was calculated per 1 µL, which was the volume used as template in each TaqMan RQ-PCR assay. Tenfold serial dilutions of the transcripts were prepared from 1.92 × 10^9^/µL–1.92 × 10^0^/µL, aliquoted and stored at −80 °C until use. Dilutions from 1.92 × 10^6^/µL–1.92 × 10^2^/µL were employed to generate the standard curve and used for the TaqMan RQ-PCR assay. The sensitivity of RQ-PCR assay was determined by testing tenfold serial dilution from 1.92 × 10^9^/µL–1.92 × 10^0^/µL of the recombinant plasmid^25^.

TaqMan RQ-PCR was carried out in Multiplate Low-Profile 96-Well Unskirted PCR Plates (Bio-Rad, USA), closed with Microseal ‘B’ Adhesive Seals (Bio-Rad, USA). Amplification and detection were performed using a CFX Connect^TM^ Real-Time PCR detection system (Bio-Rad, USA) and accompanying software (Bio-Rad CFX Manager 3.1) according to the manufacturer’s instructions. The TaqMan RQ-PCR protocol was performed in a final volume of 20 μL. Each tube contained: 1 μL forward primer (10 μM), 1 μL reverse primer (10 μM), 0.4 μL fluorogenic probe (10 μM), 10 μL *TransStart* ^*®*^ Probe qPCR SuperMix (Beijing TransGen Biotechnology Co., Ltd., Beijing, China), 1 μL sample DNA and 6.6 μL ultra-pure water. Each DNA sample was assayed in triplicate. Three wells containing 1 μL ultra-pure water each were used for a nontemplate control, without any nucleic acid. Amplification and detection were performed by using the manufacturer’s standard protocols. The PCR protocol consisted of an initial step of 30 s at 94 °C, followed by 40 cycles of 5 s at 94 °C and 30 s at 55 °C. All standards (obtained from purified plasmid DNA), controls and unknown samples were run simultaneously. Data acquisition and analysis were performed with the Bio-Rad CFX Manager 3.1 software. The CNTF were determined from the *Ct* value (real-time PCR cycle number where fluorescence signal curve crosses certain threshold line^60^). For generation of standard quantization curves, the *Ct* values were plotted proportionally against the log_10_ of the number of input copies. The *Ct* values were subsequently used to calculate the amount of DNA in unknown samples by the equations of standard curves.

### Detection efficiency of molecular diagnostic marker in spiders feeding trials

To determine whether the DNA of *E*. *vitis* could be detected in the gut of the predator, separate feeding trials were performed using adult *E*. *albaria*, *X*. *ephippiatus*, *M*. *pulcherrima* and *C*. *octomaculatum* that were collected in the tea plantation. The spiders were reared individually in 20 × 100 mm glass tube with moistened sponge in the bottom to ensure high humidity. All the spiders (including males and females) for the experiment were starved for 5–7 d under greenhouse conditions at 25 ± 1 °C, 80–85% RH and L12: D12 h photoperiod prior to the start of the experiment. After starvation, individual spiders were provided one adult *E*. *vitis* and allowed to feed for 1 h. Spiders that were not observed to feed were excluded from the experiment. After feeding, we tested the detection efficiency for four spider species at post-feeding intervals of 0, 12, 24, 36, 48, 60 and 72 h. Each post-feeding interval was provided five individual spiders. After each feeding interval, spiders were placed individually in 1.5 mL microcentrifuge tubes with 100% ethanol, and stored at −80 °C, and later submitted to the DNA extraction procedure as described above. After DNA extraction, the DNA samples from each feeding interval were amplified by TaqMan RQ-PCR using the primers (ZJ-F and ZJ-R). PCR conditions and thermocycling program as described above. Each run contained a nontemplate control (without any nucleic acid), a negative control (adult *E*. *albaria*, starved for 5–7 d) and a positive control (adult *E*. *albaria* fed on one adult *E*. *vitis*). Each sample was assayed in triplicate^25^.

### Qualitative and quantitative evaluation of predation in the field

We qualitatively and quantitatively detected the DNA samples from field-caught spiders using TaqMan RQ-PCR. PCR conditions and thermocycling program as described above. Each run contained a nontemplate control (without any nucleic acid), a negative control (adult *E*. *albaria*, starved for 5–7 d) and a positive control (adult *E*. *albaria* fed on one adult *E*. *vitis*). Each sample was assayed in triplicate. The CNTF remain in the gut of spiders from field-collected samples were calculated by interpolation from the DNA standard curve amplified in the same PCR run. The positive rates of target fragment (PRTF) was calculated using the following equations: PRTF = (Number of positive spiders/Total spiders tested) × 100%. Residual minimum number of *E*. *vitis* in individual spider guts was calculated by *N*/*M* (*N* is the CNTF of field-collected individual spiders; *M* is the CNTF of individual spiders which feeding one adult *E*. *vitis* after 0 hour digestion).

### Data analysis

The temporal overlap between predators and pests was estimated by following measure, developed by Hurlbert^61^: ${Lij}={S}{\sum }_{h=1}^{S}{P}_{ih}{P}_{jh}$; where, *L*_*ij*_ is the temporal and spatial overlap measure of species *i* on species *j*, *S* is the unit number of resource sequence, *P*_*ih*_ is the proportion that resource *h* is of the total resource that species *i* utilizes and *P*_*jh*_ is the proportion that resource *h* is of the total resource that species *j* utilizes. Difference test of percentage was used to compare the PRTF of *E*. *vitis* per dominant spider species. One-way ANOVA was used to compare the residual minimum number of *E*. *vitis* per dominant spider species.

## Electronic supplementary material

Supplementary Information
