# Supplementary material for: Molecular identification of spiders preying on Empoasca vitis in a tea plantation
Source: Sci Rep. 2017 Aug 10;7:7784. doi: 10.1038/s41598-017-07668-w (PMC5552770; doi:10.1038/s41598-017-07668-w)
Supplement: Supplementary file 1 — Supplementary Information [file 41598_2017_7668_MOESM1_ESM.pdf]

Molecular identification of spiders preying on *Empoasca vitis* in a tea plantation

Ting-bang Yang[1,+], Jie Liu[1,+], Long-yu Yuan[1], Yang Zhang[1], Dai-qin Li[3], Ingi Agnarsson[2,*] & Jian Chen[1,*]

[1]Centre for Behavioural Ecology and Evolution (CBEE), and Hubei Collaborative Innovation Center for Green Transformation of Bio-Resources, College of Life Sciences, Hubei University, Wuhan, 430062, China
[2]Department of Biology, University of Vermont, Burlington, VT, USA
[3]Department of Biological Sciences, National University of Singapore, Singapore

[*]Corresponding. Jian Chen, E-mail: chen.jian_hb@foxmail.com and Ingi Agnarsson, E-mail: iagnarsson@gmail.com.
[+]These authors contributed equally to this work.

Supporting information

Additional supporting information may be found in the online version of this article:

Table S1. Statistic of the spider species in the canopies of tea.

Table S2. Population dynamics of *E. vitis* and four dominant spider species in the canopies of tea. Values presented as mean ± SD (N = 3).

Table S3. Effect of background DNA of predators on detection of *E. vitis*. Values presented as mean ± SD. CV stands for coefficients of variation. Exp1, the genomic DNA dissolved in ultra-pure water; Exp2, the genomic DNA dissolved in the genomic DNA mixture of four dominant spider species.

Figure S1. A RQ-PCR standard curve for using stock plasmids of *E. vitis*. *Ct* stands for RQ-PCR cycle number where fluorescence curve crosses threshold line. Each concentration was used in triplicates. The curve was linear over the range of concentrations used (1.92×10^2–1.92×10^6 copies/µL). The amplification efficiency was 97.6%.

Figure S2. Agarose gel electrophoresis of PCR amplified DNA of field-collected individual spiders with various dilution. Cropped gels are merged and displayed (the full-length of each uncropped gel is 11.5 cm). a: Non-diluted DNA sample; b: Diluted by 3-fold with elution buffer; c: Diluted by 6-fold with elution buffer; d: Diluted by 9-fold with elution buffer. Lanes 1: DNA marker; Lanes 2–16: adult *E. albaria* from field collection.

Table S1. Statistic of the spider species in the canopies of tea.

	Family	Species	Field-collected number (adult)	Dominance (%)
	Salticidae	*Evarcha albaria*	382	43.2
		Phintella bifurcilinea	23	2.6
		Phintella melloteei	17	1.9
		Plexippus setipes	16	1.8
		Phintella yinae	3	0.3
		Bianor hotingchiehi	1	0.1
		Carrhotus xanthogramma	1	0.1
		Phintella debilis	1	0.1
	Theridiidae	*Meotipa pulcherrima*	75	8.5
		Coleosoma octomaculatum	74	8.4
		Theridion mirabilis	20	2.3
		Phycosoma stictum	3	0.3
		Phycosoma amamiense	2	0.2
		Euryopis galeiforma	2	0.2
		Theridula gonygaster	2	0.2
		Takayus lushanensis	2	0.2
		Meotipa vesiculosa	1	0.1
	Araneidae	*Araneus ejusmodi*	25	2.8
		Neoscona mellotteei	22	2.5
		Neoscona adianta	6	0.7
		Cyclosa atrata	1	0.1
		Hypsosinga pygmaea	1	0.1
		Hypsosinga sanguinea	1	0.1
		Neoscona theisi	1	0.1
		Neoscona kunmingensis	1	0.1
	Thomisidae	*Xysticus ephippiatus*	95	10.7
		Ebrechtella tricuspidata	3	0.3
		Monaeses aciculus	3	0.3
		Xysticus croceus	2	0.2
		Xysticus hedini	2	0.2
		Thomisus labefactus	1	0.1
		Xysticus cristatus	1	0.1
		Boliscus tuberculatus	1	0.1
		Oxytate hoshizuna	1	0.1
	Clubionidae	*Clubiona deletrix*	33	3.7
		Clubiona corrugata	1	0.1
	Tetragnathidae	*Tetragnatha squamata*	7	0.8
		Pachygnatha tenera	2	0.2
	Gnaphosidae	*Zelotes asiaticus*	5	0.6
		Zelotes tongdao	2	0.2

	Oxyopidae	*Oxyopes sertatus*	26	2.9
		Oxyopes javanus	2	0.2
	Lycosidae	*Pardosa sumatrana*	1	0.1
		Pardosa laura	1	0.1
	Linyphiidae	*Allomengea adornata*	1	0.1
	Miturgidae	*Prochora praticola*	1	0.1
	Titanoecidae	*Nurscia albofasciata*	1	0.1
	Agelenidae	*Agelena labyrinthica*	1	0.1
	Trachelidae	*Trachelas japonicus*	7	0.8
	Eutichuridae	*Cheiracanthium japonicum*	2	0.2
Total	15	50	885	

Table S2. Population dynamics of *E. vitis* and four dominant spider species in the canopies of tea. Values presented as mean ± SD (N = 3).

Month	Field-collected number (juvenile and adult)				
	E. vitis	***E. albaria***	***X. ephippiatus***	***M. pulcherrima***	***C. octomaculatum***
Jan. 2013	204.7 ± 23.7	3.0 ± 1.0	0	0	3.3 ± 3.1
Feb. 2013	184.3 ± 30.7	5.0 ± 1.0	0	2.7 ± 0.6	1.3 ± 2.3
Mar. 2013	243.0 ± 53.5	9.3 ± 2.5	3.3 ± 1.2	2.3 ± 0.6	16.0 ± 4.8
Apr. 2013	370.0 ± 113.5	11.7 ± 6.4	4.0 ± 1.0	1.3 ± 1.5	9.3 ± 5.5
May 2013	684.0 ± 35.6	18.0 ± 2.0	5.7 ± 0.6	4.7 ± 5.0	8.0 ± 7.1
Jun. 2013	605.0 ± 118.2	32.0 ± 12.0	9.0 ± 5.3	4.7 ± 1.2	3.3 ± 0.6
Jul. 2013	488.3 ± 34.6	61.7 ± 6.0	9.7 ± 4.7	10.0 ± 3.0	2.3 ± 0.6
Aug. 2013	513.3 ± 168.0	86.3 ± 11.9	8.7 ± 3.1	4.0 ± 3.6	1.0 ± 1.7
Sep. 2012	664.7 ± 72.0	45.3 ± 10.7	6.0 ± 2.0	4.0 ± 2.6	3.7 ± 2.1
Oct. 2012	344.7 ± 191.1	24.7 ± 6.7	9.7 ± 4.6	1.0 ± 1.7	3.7 ± 3.5
Nov. 2012	193.7 ± 31.2	10.3 ± 2.9	3.7 ± 1.2	2.0 ± 2.6	1.3 ± 2.3
Dec. 2012	149.7 ± 29.3	7.0 ± 1.7	0	1.3 ± 1.5	3.3 ± 5.8

Table S3. Effect of background DNA of predators on detection of *E. vitis*. Values presented as mean ± SD. CV stands for coefficients of variation. Exp1, the genomic DNA dissolved in ultra-pure water; Exp2, the genomic DNA dissolved in the genomic DNA mixture of four dominant spider species.

Dilution of genomic DNA of *E. vitis*	Exp 1		Exp 2		Inter-assay (between Exp1 and Exp2)	
	Ct	**CV (%)**	***Ct***	**CV (%)**	***Ct***	**CV (%)**
1/10	24.34 ± 0.42	1.74	24.08 ± 0.18	0.74	24.21 ± 0.19	0.78
1/100	27.17 ± 0.34	1.25	26.81 ± 0.41	1.51	26.99 ± 0.26	0.95
1/1000	29.75 ± 0.46	1.55	29.68 ± 0.14	0.46	29.71 ± 0.05	0.17
1/10000	32.93 ± 0.34	1.04	33.39 ± 1.33	3.98	33.16 ± 0.32	0.98

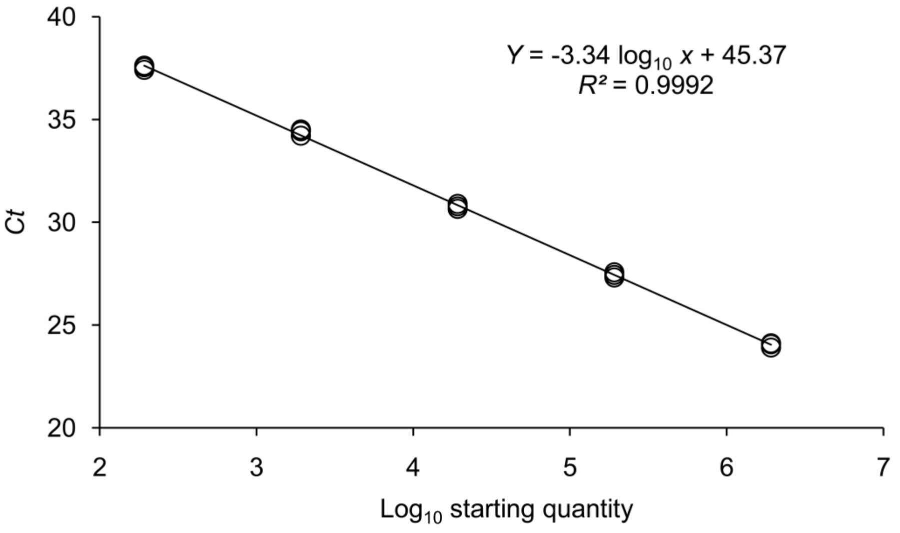

Figure S1. A RQ-PCR standard curve for using stock plasmids of *E. vitis*. *Ct* stands for RQ-PCR cycle number where fluorescence curve crosses threshold line. Each concentration was used in triplicates. The curve was linear over the range of concentrations used (1.92×10^2–1.92×10^6 copies/μL). The amplification efficiency was 97.6%.

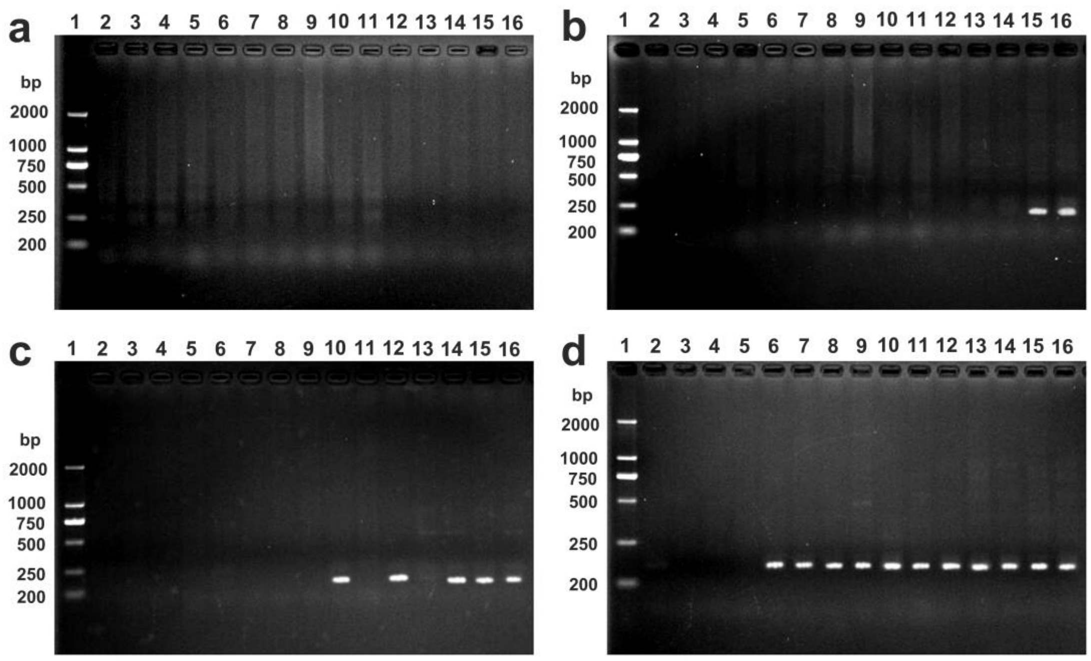

Figure S2. Agarose gel electrophoresis of PCR amplified DNA of field-collected individual spiders with various dilution. Cropped gels are merged and displayed (the full-length of each uncropped gel is 11.5 cm). a: Non-diluted DNA sample; b: Diluted by 3-fold with elution buffer; c: Diluted by 6-fold with elution buffer; d: Diluted by 9-fold with elution buffer. Lanes 1: DNA marker; Lanes 2–16: adult *E. albaria* from field collection.
